# Cross-Linked Self-Assembling Peptides and Their Post-Assembly Functionalization via One-Pot and In Situ Gelation System

**DOI:** 10.3390/ijms21124261

**Published:** 2020-06-15

**Authors:** Raffaele Pugliese, Fabrizio Gelain

**Affiliations:** 1 Tissue Engineering Unit, Institute for Stem Cell Biology, Regenerative Medicine and Innovative Therapies-ISBReMIT, Fondazione IRCCS Casa Sollievo della Sofferenza, 71013 San Giovanni Rotondo (FG), Italy; r.pugliese@operapadrepio.it; 2 Center for Nanomedicine and Tissue Engineering (CNTE), ASST Grande Ospedale Metropolitano Niguarda, 20162 Milan, Italy

**Keywords:** self-assembling peptides, supramolecular hydrogels, cross-linking, EDC, sulfo–NHS, functional motifs, rheology, mechanical properties

## Abstract

Supramolecular nanostructures formed through peptide self-assembly can have a wide range of applications in the biomedical landscape. However, they often lose biomechanical properties at low mechanical stress due to the non-covalent interactions working in the self-assembling process. Herein, we report the design of cross-linked self-assembling peptide hydrogels using a one-pot in situ gelation system, based on 1-ethyl-3-[3-dimethylaminopropyl] carbodiimide/N-hydroxysulfosuccinimide (EDC/sulfo–NHS) coupling, to tune its biomechanics. EDC/sulfo–NHS coupling led to limited changes in storage modulus (from 0.9 to 2 kPa), but it significantly increased both the strain (from 6% to 60%) and failure stress (from 19 to 35 Pa) of peptide hydrogel without impairing the spontaneous formation of β-sheet-containing nano-filaments. Furthermore, EDC/sulfo–NHS cross-linking bestowed self-healing and thixotropic properties to the peptide hydrogel. Lastly, we demonstrated that this strategy can be used to incorporate bioactive functional motifs after self-assembly on pre-formed nanostructures by functionalizing an Ac-LDLKLDLKLDLK-CONH_2_ (LDLK12) self-assembling peptide with the phage display-derived KLPGWSG peptide involved in the modulation of neural stem cell proliferation and differentiation. The incorporation of a functional motif did not alter the peptide’s secondary structure and its mechanical properties. The work reported here offers new tools to both fine tune the mechanical properties of and tailor the biomimetic properties of self-assembling peptide hydrogels while retaining their nanostructures, which is useful for tissue engineering and regenerative medicine applications.

## 1. Introduction

Mechanobiology is drawing widespread attention for its potential value in the field of biomaterial design for tissue engineering and regenerative medicine [1,2].

Cells are sensitive to the mechanical properties of the extracellular matrix (ECM) in both physiological and pathological conditions (such as stroke, brain trauma, spinal cord injuries, cartilage damage, and tumors) [3,4,5]; it is becoming increasingly clear that the mechanical properties of the ECM are critical in directing cell fate, homeostasis, and survival [6,7,8].

Hence, synthetic but bio-inspired nanomaterials designed to mimic the ECM should aim to recapitulate most of the features of the native ECM. Scaffold nanoarchitecture and mechanical properties should be tuned in accordance with the targeted tissue and to achieve the precise control of cellular behavior [9]. Just like the ECM, the chemical structure of the biomaterial should concurrently contain non-covalent and covalent chemical bonds, bringing, respectively, infinite and finite lifetimes of interactions [10] to obtain a reversibly dynamic matrix. Moreover, the method for incorporating biological epitopes (also named functional motifs) into the material should be applicable to a broad array of bioactive molecules (from small peptides to large proteins) before and after synthesis/processing in order to add new tools to maximize material–cell interactions [11].

Biomaterials based on peptide self-assembly meet some of these criteria [12,13]. However, despite the fact that self-assembling peptides (SAPs) can self-assemble into high-aspect-ratio nanofibers resembling nanofibrous ECMs [14,15,16,17,18], the practical application of such nanomaterials has been limited due to their intrinsic instability, low-performance, and low-strain/stress response [19]. Further, the incorporation of functional motifs into SAP nanostructures has generally been limited to bioactive peptides tethered to the C- or N-terminal of an SAP sequence during solid-phase peptide synthesis [20,21,22,23,24,25,26,27,28]. Additionally, the presence of functional motifs, bringing additional hydrophobic and charged interactions, could potentially influence the self-assembly process of the SAP molecules, resulting in the formation of an altered nanostructures [29] and consequently posing limits to their potential functionalization [20,30]. Therefore, until now, the multiple functionalizations of SAPs with different bioactive cues have been predominantly obtained via the self-assembly of differently functionalized SAPs sharing the same self-assembling sequence [19,31,32].

Recently, our group pioneered the use of cross-linked peptide hydrogels with tuned and highly increased mechanical features suiting the needs of different regenerative medicine applications that, at the same time, effectively display bioactive motifs at their nanostructures [33,34]. Other groups have also utilized covalent capture to design more robust SAP-biomaterials under a variety of conditions [35,36,37]. However, these synthetic methods are relatively cumbersome and require long reaction times (up to 24 h). In contrast, physical cross-linking can enhance the mechanical properties of self-assembled fibrillar networks by influencing specific intermolecular interactions that modulate fiber intertwining. For example, calcium-mediated ionic bridges can form stronger intra- and inter-fiber cross-links among SAP molecules, and, as a result, cross-linked hydrogels can withstand higher strains [35].

**Figure 1:**
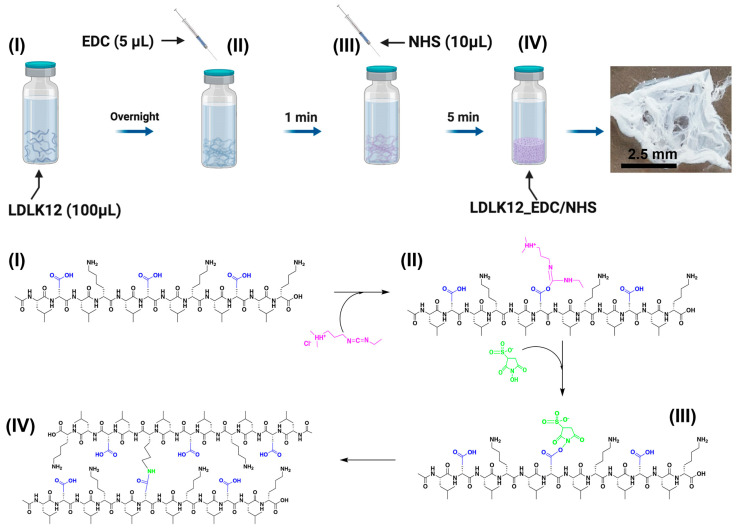
EDC/sulfo–NHS (1-ethyl-3-[3-dimethylaminopropyl] carbodiimide/*N*-hydroxysulfosuccinimide) cross-linking reaction. Schematic illustration and chemical reaction of the one-pot and in situ LDLK12 (Ac-LDLKLDLKLDLK-CONH_2_) cross-linking based on EDC/sulfo–NHS coupling: (**I**) LDLK12, (**II**) O-acylisourea intermediate LDLK12 peptide, (**III**) sulfo–NHS-modified LDLK12 peptide, and (**IV**) EDC/sulfo–NHS cross-linked LDLK12 peptides. In the schematic representation of the amide bond formation, the LDLK12 peptide is shown in black, the carboxyl group is shown in blue, EDC is shown in magenta, and sulfo–NHS is shown in light green.

Here, we report the use of 1-ethyl-3-[3-dimethylaminopropyl] carbodiimide/*N*-hydroxysulfosuccinimide (EDC/sulfo–NHS) coupling to readily cross-link LDLK12 (Ac-LDLKLDLKLDLK-CONH_2_) SAP molecules (Figure 1 and Figure S1). The cross-linked LDLK12 features enhanced mechanical features and can be further cross-linked (decorated) with bioactive motifs to expand its range of applications. EDC/sulfo–NHS is one of the most commonly used carbodiimides, and it catalyzes the formation of amide bonds between carboxyl and primary amine groups. Its broad use is derived from its high solubility in water and its ease of removal of the byproduct [38].

By using a one-pot and in situ gelation system, we demonstrated, for the first time, that EDC/sulfo–NHS specifically reacts with LDLK12-assembled nanostructures, enhancing their mechanical properties without altering the spontaneous formation of β-sheet-containing nanofilaments. Lastly, we investigated the use of a post-assembly modification of LDLK12 nanostructures using EDC/sulfo–NHS as an alternative approach to add bioactive functional motifs after self-assembling took place, i.e., for all jellified SAP hydrogels. We carried out such a post-assembly functionalization of nanofibers by using a KLPGWSG [22] phage display-derived epitope as a bioactive cue.

This versatile approach may be potentially applied to biomaterials containing aspartic acid (D) or glutamic acid (E), and lysine (K) residues to enhance their biomechanics and biomimetic properties, hence leading to a new precious tool for biomedical applications.

## 2. Results and Discussion

### 2.1. EDC/Sulfo–NHS Cross-Linked Peptide Preparation

Water-soluble EDC carbodiimide reacts with carboxylic acids and forms reactive *O*-acylisourea intermediates, which are then linked to a nucleophile (i.e., a primary amine) to create an amide bond. However, *O*-acylisourea intermediates are labile in the presence of polar solvents and must react immediately after dissolution in water. Indeed, oxygen atoms from water can also act as a nucleophile, cleaving off the intermediate and releasing isourea, thus inactivating EDC molecules [39].

To improve the coupling efficiency, EDC can be used in conjunction with sulfo–NHS to form an active ester with the carboxylic acid. This type of intermediate ester is hydrophilic, stable, and hydrolyzes relatively slowly in water (circa 4–5 h), offering an advantage for coupling reactions. In the presence of amine nucleophiles (e.g., lysine side-chains in peptide molecules), the sulfo–NHS ester is rapidly hydrolyzed, allowing for the formation of an amide bond.

To investigate the EDC/sulfo–NHS coupling reaction, we selected the previously characterized SAP LDLK12 [17,19,40], which spontaneously self-assembles into nanofibers. When pH is triggered from acidic (pH~4.5) to neutral values (pH~7.4), nanofibers in the LDLK12 solution form bulk hydrogels that act as a biodegradable artificial ECM with proven neuroregenerative potential in vitro and in vivo [41].

The LDLK12 peptide was dissolved in distilled water (pH 5.5) to achieve a final concentration of 1% (*w*/*v*), and it was then left overnight at +4 °C in order to guarantee the spontaneous formation of pre-assembled nanofibers. Afterwards, EDC (0.2 M, 5 μL) in Dulbecco’s phosphate-buffered saline (DPBS) (pH 7.4) was added dropwise to the pre-assembled peptide bundles to obtain *O*-acylisourea intermediate peptides. Immediately, sulfo–NHS (0.2 M, 10 μL) was added to the EDC pre-activated peptide solution. Sulfo–NHS promptly reacted with the water-exposed lysine in the peptide bundles, thus forming a stable amide cross-link among the pre-assembled peptide molecules (Figure 1). When the sulfo–NHS solution was added to the solution of *O*-acylisourea pre-activated SAP, we observed the formation of self-supporting cross-linked scaffolds in less the 5 min.

Coupling yield was minimal when EDC/sulfo–NHS was added to SAPs immediately after their solubilization in water, while, after the chosen overnight stay, cross-linking showed a rapid peptide coupling after EDC/sulfo–NHS addition. This suggests an interesting correlation between the degree of coupling and the physico-chemical conditions of peptide molecules.

Since this reaction takes place in situ, at physiological conditions (pH 7.4), relatively fast, and without the need of external stimuli (such as temperature or ionic strength), it could be useful as filler in traumatic brain injury (TBI) [42,43], acute spinal cord injury (SCI) [41,44,45,46], or as sprayable hemostatic solution in combination with common clotting bandages during uncontrolled bleeding in surgeries [47].

### 2.2. Mechanical Properties of Peptide Nanostructures

**Figure 2:**
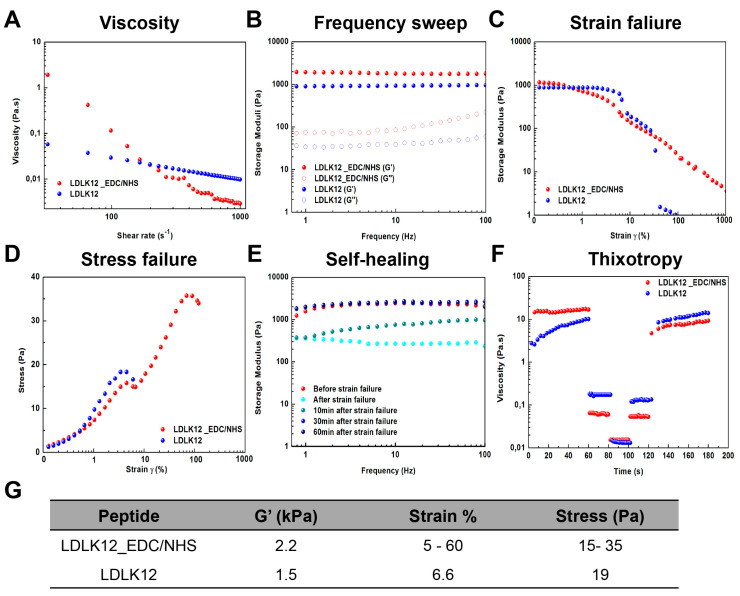
Rheological studies to evaluate the mechanical properties of the LDLK12 hydrogels. (**A**) Viscosity measurements at increasing shear rate of the wildtype and cross-linked LDLK12 peptides; (**B**) frequency-dependent oscillatory rheology (0.1–100 Hz) of the LDLK12 hydrogels (1% *w*/*v*) at 1% strain (within the linear viscoelastic regime (LVR)); (**C**) strain– and (**D**) stress–sweep analyses of LDLK12 hydrogels at a constant frequency of 1 Hz (cross-linked LDLK12 exhibited an unusual two-step shear strain–stress behavior); (**E**) self-healing of cross-linked LDLK12; (**F**) thixotropy test of standard and cross-linked LDLK12 peptide solutions; and (**G**) average values of the storage modulus (G′), strain, and stress.

To investigate the impact of EDC/sulfo–NHS cross-linking, rheological studies were conducted to evaluate the mechanical properties of the LDLK12 hydrogel. First, we analyzed the viscosity of peptide solutions (Figure 2A). Both SAPs with (red dots) and without (blue dots) EDC/sulfo–NHS cross-linking displayed non-Newtonian shear-thinning behavior with a decrease of viscosity that was concomitant with the shear-rate increase. Even if the cross-linked LDLK12 showed an increased viscosity (1.9 Pa.s) compared to the standard LDLK12 (0.06 Pa.s), both hydrogels had negligible differences at higher shear rate values (500–1000 s^−1^). The non-Newtonian shear-thinning behavior of both SAPs was also confirmed by assessing the shear stress (σ) trend alongside shear-rate increments (Figure S2).

Next, we investigated the storage and loss moduli (G′ and G″, respectively) in the function of angular frequency (1–100 Hz). The G′ value (full dots) of both peptides was found to be higher than G″ (empty dots), indicating the formation of a hydrogel with a predominant solid-elastic behavior (Figure 2B). However, the cross-linked SAP displayed a small change in elastic shear modulus (2.2 kPa) compared to the standard LDLK12 (1.5 kPa), which still indicated a slight increment of the gelation propensity of the cross-linked LDLK12 due to the EDC/sulfo–NHS coupling.

Since recent work has demonstrated that G′ is not the sole determining factor in cell mechanobiology [1,48,49,50,51], so the shear strain–stress response was also determined to assess the cross-linked LDLK12 failure when subjected to a linear strain–stress progression. Strain–sweep experiments (Figure 2C) demonstrated a wide linear viscoelastic regime (LVR) of the cross-linked LDLK12 (red dots) and an unusual bi-modal failure at 5% and 60% of strain, respectively, that had never been observed before in SAP-based biomaterials. Indeed, standard LDLK12 (blue dots), belonging to fragile and soft-hydrogels, showed a one-step strain failure at 6.6%. As a consequence, in its stress–failure curves (Figure 2D), the cross-linked LDLK12 displayed a two-step unusual stress–failure process. This characteristic only being observed on the cross-linked LDLK12 suggests that the two-step shear strain–stress behavior may be strictly dependent on the EDC/sulfo–NHS coupling and could be correlated with the morphological transitions caused by the supramolecular rearrangement of LDLK12 reacted with EDC/sulfo–NHS. Moreover, it is worth noting that these stain–stress rates fell within a regime that has been shown to be conducive to matrix reorganization [48,49,52], implying that the EDC/sulfo–NHS coupling system could be suited for 3D cell cultures.

We also observed a spontaneous self-healing propensity of the cross-linked LDLK12 (Figure 2E). After a large strain failure (γ = 1,000%; cyan dots), the G′ of the cross-linked LDLK12 returned to its original values after 30 min (blue dots), demonstrating a recovery of the mechanical properties in the LVR. A possible explanation for this observation may be the simultaneous presence of non-covalent and covalent bonds, which led to the formation of a dynamic supramolecular system capable of self-healing without the need of external stimuli. On the contrary, as we previously described [53], when exerting larger amplitude oscillatory deformation (γ > 100%), LDLK12-based SAPs show a negligible mechanical recovery, implying that they lose parts of mechanical stability because of their weak and brittle nature.

Since injectable hydrogels have gained increasing amounts of attention in the fields of tissue engineering and drug, cells, or growth factor delivery due to their minimally invasive way of delivery [54,55,56], to evaluate the propensity of LDLK12-materials to recover their initial viscosity after injection, the thixotropy of all solutions was investigated ex vivo.

In this test, the injection conditions were simulated through a series of constant shear rate tests (see Materials and Methods for further details). Both SAPs with (red dots) and without (blue dots) EDC/sulfo–NHS cross-linking exhibited a fast recovery after injection simulation (Figure 2F). This fast viscosity recovery hinted a space-filling propensity of all solutions after injection, highlighting that both LDLK12 scaffolds could readily provide a good fit and interface between the hydrogel and damage tissues.

Our data demonstrated that the EDC/sulfo–NHS coupling could be applied to the LDLK12 systems without hampering their predominant solid-elastic behavior (Figure 2G). In addition, the cross-linked LDLK12 peptide demonstrated a wide LVR with an unusual two-step shear strain–stress behavior, which was unmatched by other SAP scaffolds, typically relying on non-covalent interactions for their biomechanics.

### 2.3. Supramolecular Organizations of Peptide Nanostructures

To gain insights into the supramolecular and global arrangement of the cross-linked peptide hydrogel, we used a Thioflavin-T (ThT)-binding assay, FT-IR spectroscopy, circular dichroism (CD), and atomic force microscopy (AFM) morphological analysis.

**Figure 3:**
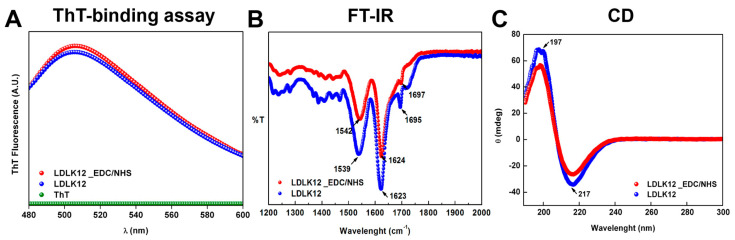
Supramolecular organizations of assembled peptide nanostructures. (**A**) Thioflavin-T (ThT)-binding assay of LDLK12 hydrogels showing typical amyloid-binding emission signal (centered at ∼500 nm); no signal was observed for ThT alone. (**B**) FT-IR spectra of the wildtype and cross-linked LDLK12 peptides with characteristic β-sheet peaks. (**C**) Circular dichroism (CD) spectra of both self-assembling peptides (SAPs) with and without EDC/sulfo–NHS cross-linking suggesting the presence of β-sheet secondary structures.

A ThT-binding assay, an amyloid-specific fluorescent dye [57,58,59] (see Materials and Methods), was used to examine the amyloidogenic nature of the cross-linked LDLK12 fibers (Figure 3A). Staining the fibers with ThT resulted in high fluorescence levels with a typical amyloid-binding emission signal (centered at ∼500 nm): this highlighted that both SAPs with (red dots) and without (blue dots) EDC/sulfo–NHS cross-linking featured a similar amyloid-like nature.

To study the secondary structure of the peptide in solution, we carried out FT-IR spectroscopy tests (Figure 3B). FT-IR spectra exhibited a sharp Amide I band at ∼1630 cm^−1^ with a shoulder at ∼1695 cm^−1^, indicating predominantly anti-parallel β-sheet features. The band at ∼1540 cm^−1^ in the Amide II region also confirmed the β-sheet aggregation of both peptide samples. In the cross-linked LDLK12, these peaks were slightly red-shifted compared to the β-sheet signal of the standard LDLK12; this might have been ascribable to the interference of sulfo–NHS in the formation of the amide bonds between the *O*-acylisourea pre-activated SAP chains.

To explore the effect of EDC/sulfo–NHS on the formation of β-sheet conformation of LDLK12 assemblies, CD spectra were recorded (Figure 3C). Positive and negative peaks at, respectively, 197 and 217 nm, typical of β-sheet rich structures, were observed for both SAPs with (red dots) and without (blue dots) EDC/sulfo–NHS cross-linking. No assembly was observed for EDC/sulfo–NHS alone (Figure S3).

**Figure 4:**
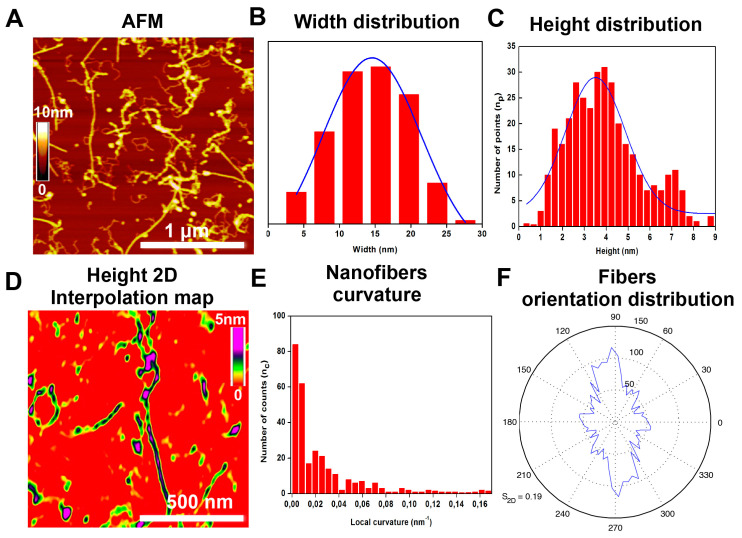
Morphological organizations of assembled peptide nanostructures. (**A**) Atomic force microscopy (AFM) images of the cross-linked LDLK12 that self-organize into elongated and unbranched fibers; (**B**) width fibers distribution and (**C**) height profiles of cross-linked peptide fibers; (**D**) 2D interpolation heights map of the cross-linked LDLK12 showing an average value of 3.65 nm; (**E**) cross-linked nanofibers curvature profile; and (**F**) orientation distribution of the cross-linked LDLK12 fibers along one direction.

AFM was used to elucidate the fiber morphology of the cross-linked LDLK12. After EDC/sulfo–NHS cross-linking, the LDLK12 peptide revealed the presence of entangled fibers (Figure 4A). The cross-linked peptide adopted an elongated and unbranched nanofiber network morphology with a width of ~15 ± 4 nm (Figure 4B). The height distribution ranged from 1.06 to 8.76 nm (Figure 4C) and peaked at 3.65 ± 0.7 nm, as depicted in the 2D interpolation maps (Figure 4D and Figure S4).

In addition, we characterized the curvature of the cross-linked LDLK12 nanofibers (Figure 4E). The AFM images of the cross-linked LDLK12 revealed a wide range of nanofibrils curvature values, with a maximum curvature value of 0.17 nm^−1^. This was unusual, since standard LDLK12-based fibers usually appear rigid and flat [19]. These data are in good agreement with the rheological analysis and could explain the reason for the large shear strain/stress resistance of the cross-linked LDLK12 bestowed by cross-linking with EDC/sulfo–NHS. Lastly, the cross-linked LDLK12 fibrils showed an orientation distribution with a clear peak along one direction (Figure 4F), and they also exhibited a strongly aligned nematic fibril domain [60] (Figure S5).

In summary, the spectroscopic characterization (ThT-binding assay and FT-IR) and AFM analysis confirmed the amyloid-like nature and self-assembling propensity into β-sheets of the cross-linked LDLK12, suggesting that the introduction of EDC/sulfo–NHS does not impair the formation of β-sheet-containing nano-filaments, but it does lead to the formation of mainly aligned curved supramolecular nanostructures that are more resistant to shear strains.

### 2.4. Post-Assembly Functionalization of KLPGWSG Peptide to the Surface of SAP Nanofibers

Next, we sought to extend the scope of the EDC/sulfo–NHS coupling to the functionalization of SAP nanofibers. To demonstrate that the EDC/sulfo–NHS reaction can be used to ligate bioactive cues to the LDLK12 nanofibers, we utilized a phage display-derived KLPGWSG bioactive peptide that has been shown to interact with neural stem cells and to significantly shift their differentiation toward the neuronal phenotype. [22] Indeed, we previously demonstrated that, by using BLAST analysis, the major consensus sequences for KLPGWSG are found on three proteins known to affect stem cells behaviors: namely Notch1, Dll4, and MEGF10 [22].

**Figure 5:**
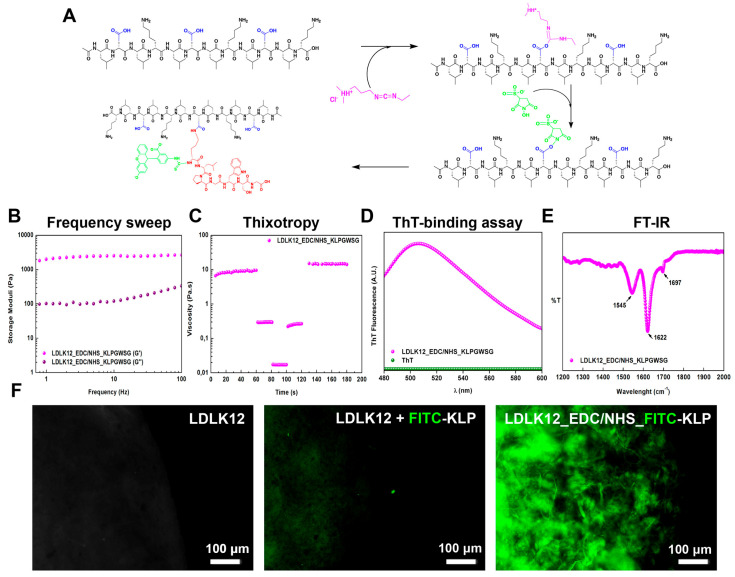
Post-assembly functionalization of KLPGWSG peptide. (**A**) Reaction scheme of the EDC/sulfo–NHS-mediated conjugation of fluorescein isothiocyanate (FITC)-KLPGWSG to LDLK12. The LDLK12 peptide is shown in black, FITC-KLPGWSG is shown in red, the carboxyl group is shown in blue, EDC is shown in magenta, and sulfo–NHS is shown in light green. (**B**) Dynamic frequency sweep of phage-derived KLPGWSG conjugated to the surface of LDLK12 peptides, recorded as a function of angular frequency (0.1–100 Hz) at a fixed strain of 1%; (**C**) thixotropy test of KLPGWSG post-assembly functionalized LDLK12; (**D**) ThT-binding assay showing a typical amyloid-binding emission signal (ThT alone was used as blank); (**E**) FT-IR spectrum of the LDLK12 peptide after the EDC/sulfo–NHS reaction with KLPGWSG showing a predominant β-sheet secondary structure; and (**F**) fluorescence micrographs of LDLK12 nanofibers reacted with FITC-KLPGWSG. Standard LDLK12, as well as LDLK12 and FITC-KLPGWSG without EDC/sulfo–NHS conjugation, were used as controls.

The EDC/sulfo–NHS post-assembly reaction was performed upon the simple mixing of water-soluble KLPGWSG (see Materials and Methods for further details) on the top surface of the pre-activated EDC/sulfo–NHS-assembled LDLK12 peptide hydrogel (Figure 5A). The post-assembly conjugation of the KLPGWSG molecule added highly hydrophobic aliphatic side-chains and one positively charged lysine residue to the LDLK12 fibers, resulting in an increase in surface charge and in a shift of the isoelectric point (from pH 7.19 to 10.19).

KLPGWSG-functionalized LDLK12 hydrogels were analyzed using oscillatory stress rheology. In the gel phase, phage-derived functionalized LDLK12 showed an almost unchanged G′ profile along the tested frequency range (Figure 5B), and the thixotropic and space-filling propensity of the LDLK12 based-hydrogel was still preserved (Figure 5C).

Again, the amyloidogenic nature and the secondary structures of nanofibers functionalized with KLPGWS were investigated using the ThT-binding assay, FT-IR, and CD spectroscopy. The ThT-binding assay exhibited a typical amyloid-binding emission signal centered at ∼500 nm (Figure 5D), and the FT-IR spectrum of the LDLK12 peptide after the EDC/sulfo–NHS reaction with the KLPGWSG peptide suggested a predominant β-sheet secondary structure, with a minimal change caused by the conjugation of epitope (Figure 5E). Additionally, CD spectra displaying positive and negative peaks at, respectively, 200 and 217 nm (Figure S6) confirmed the presence of β-sheet secondary structures in the LDLK12 peptide after EDC/sulfo–NHS cross-linking with KLPGWSG. In contrast, the KLPGWSG alone showed an unstructured conformation with a minor negative band at 196 nm.

These results indicated that the presence of the KLPGWS epitope on the LDLK12 molecules did not influence their self-assembly process, biomechanics, and resulting secondary structures.

To confirm that the ligated KLPGWSG epitope was present on the surface of the LDLK12 nanofibers, we added a fluorescein isothiocyanate (FITC) group to the N-terminus of the KLPGWSG (see Materials and Methods) and used EDC/sulfo–NHS to attach this peptide to the LDLK12 nanofibers. A higher fluorescence intensity was observed following the EDC/sulfo–NHS-mediated conjugation of the FITC-KLPGWSG peptide to LDLK12 nanofibers (Figure 5F). Conversely, adding the same bioactive peptide without EDC/sulfo–NHS conjugation produced a significantly lower fluorescence intensity (Figure S7), indicating that the observed fluorescence was due to the retention of the cross-linked FITC-KLPGWSG by the specific EDC/sulfo–NHS reaction with LDLK12 and not a non-specific adsorption to SAP nanofibers.

In summary, this post-assembly functionalization approach mediated by EDC/sulfo–NHS can be applied to any primary amine-containing SAPs. It is possible to combine different bioactive and fluorescent peptides to potentially decorate SAP hydrogel surfaces with multiple functions (e.g., for sensing, adhesion, or cell signaling) while maintaining their desirable structural, biomechanical, and biocompatibility properties typical of SAP biomaterials.

## 3. Materials and Methods

### 3.1. Materials

All reagents and solvents used for the peptide synthesis and characterizations were purchased from commercial sources and used without further purification.

### 3.2. Peptide Synthesis

Peptides were synthesized using a CEM Liberty-Discovery microwave-assisted peptide synthesizer and a Rink amide 4-methyl-benzhydrylamine resin (0.5 mmoL g^−1^ substitution) to tether the nascent peptide during synthesis. For the addition of each amino acid, Fmoc-protected amino acids were dissolved at 0.2 M in dimethylformamide (DMF). Fmoc removal was accomplished using a solution of 20% (*v*/*v*) 4-methylpiperidin in DMF. The 0.5 M *N,N,N′,N′-*tetramethyl-O-(1H-benzotriazol-1-yl)uronium hexafluorophosphate (HBTU) in DMF and 2 M *N,N-*diisopropylethylamine (DIEA) in *N*-methyl-2-pyrrolidone (NMP) were used as activator and activator base solutions, respectively. Resin cleavage was accomplished by adding 95% trifluoroacetic acid (TFA), 2.5% triisopropylsilane (TIS), and 2.5% H_2_O. Raw peptides were subsequently purified via the Waters binary HPLC apparatus, and the molecular weight of each peptide was identified via single quadrupole mass detection (Waters LC-MS Alliance-3100) (see Figure S8).

### 3.3. Fluorescein Isothiocyanate (FITC) Labeling

KLPGWSG (1 equivalent) and FITC (1.2 equivalents) were dissolved in a Borate buffer (pH 9) and dimethyl sulfoxide (DMSO), respectively, as previously reported [22]. The reaction mixture was stirred for 16 h at room temperature. Upon the completion of conjugation, the resulting solution was subjected to HPLC purification, and the collected fractions were lyophilized (see Figure S8).

### 3.4. Cross-Linked Peptide Preparation

Lyophilized LDLK12 was dissolved in distilled water (GIBCO^®^) to achieve a final concentration of 1% (*w*/*v*), and it was then stored at +4 °C overnight. For the cross-linking reaction, EDC (0.2 M, 5 μL) in 1X DPBS (Ca^2+^/Mg^2+^ free) was added dropwise to the peptide sample (100 μL) and vigorously stirred for 2 min. Then, sulfo–NHS (0.2 M, 10 μL) was added to the solution to form stable amide cross-links among the pre-assembled peptide molecules (Figure S9A). Unreacted sulfo–NHS can be quenched via the addition of hydroxylamine (1 M).

For the post-assembly functionalization, water-soluble FITC-KLPGWSG (1% *w*/*v*, 20 μL) was added on the top surface of the pre-activated EDC/sulfo–NHS-assembled LDLK12 peptide and then allowed to react for 15 min at room temperature (Figure S10). The reaction was quenched by adding 2-mercaptoethanol and distilled water to inactivate the EDC and to remove unbound FITC-KLPGWSG peptides.

### 3.5. Mechanical Testing

All rheology tests were performed on a stress/rate-controlled AR-2000ex Rheometer (TA Instruments,159 Lukens Drive, New Castle, DE 19720) equipped with a truncated cone-plate geometry (acrylic truncated diameter, 20 mm; angle, 1°; truncation gap, 34 μm). All measurements were obtained at 25 °C using a Peltier cell in the lower plate of the instrument to control the temperature during each test. All samples were tested one day after dissolution. Viscosity was measured using a flow step program at an increasing shear rate to evaluate the non-Newtonian behavior of the peptides. To evaluate the storage (G′) and loss (G″) moduli increases, frequency sweep experiments were recorded as a function of angular frequency (0.1–100 Hz) at a fixed strain of 1%. Stress–strain sweeps were performed on samples from 0.01% to a maximum strain of 1000% to determine the limit of the LVR and, therefore, the maximum stress–strain to which the sample could be tested. To test the thixotropy of peptides, shear-thinning tests were performed by a series of peak hold tests in which shear rates were kept constant, as we previously reported [19]. Briefly, firstly a shear rate of 0.01 s^−1^ was applied for 60 s, and then a shear rate of 5.3 s^−1^ was applied for 20 s to simulate the shear rate inside the syringe barrel. Subsequently, a high shear rate of 1000 s^−1^ was applied for 20 s to simulate the purge injection of the solution. Afterwards, a shear rate of 5.3 s^−1^ (20 s) was applied again, thus mimicking the flow of peptide solution out of the needle. Lastly, a shear rate of 0.01 s^−1^ was performed in order to simulate the low shear condition of the solution during injection. Data were processed using the Origin™ 8 software (OriginLab Corporation, Northampton, MA 01060, USA).

### 3.6. Thioflavin T (ThT) Spectroscopy Assay

The ThT analysis of assembled peptides was performed to assess the presence of amyloidogenic fibril structures. Peptide samples (40 μM) were mixed with the ThT solution (20 μM) and stirred for 4 min, as previously reported [19]. ThT fluorescence intensity was recorded using an Infinite M200 PRO plate reader (Tecan) with λ_ex_ = 440 nm (5 nm bandpass) and λ_em_ = 482 nm (10 nm bandpass) over 60 s at 25 °C. Measurements were normalized over ThT-alone fluorescence and processed with the Origin™ 8 software.

### 3.7. Fourier Transform Infrared Spectroscopy (FT-IR)

The FT-IR spectra of the assembled nanostructures were acquired in attenuated total reflection (ATR) using a PerkinElmer Spectrum 100 spectrometer. Twenty acquisitions were recorded for each spectrum using the following conditions: 4 cm^−1^ spectrum resolution, 25 kHz scan speed, 1000-scan co-addition, and triangular apodization. All the obtained spectra were reported after ATR correction, smoothing, and automatic baseline correction using the Origin™ 8 software.

### 3.8. Circular Dichroism spectroscopy (CD)

The CD spectra of all the peptides were recorded on a Jasco J-815 (Jasco Corp., Tokyo, Japan) spectropolarimeter using a 0.1 mm quartz cuvette. Spectra were collected in the 180–300 nm spectral range and averaged over three scans at room temperature. All the scans were carried out at a scan speed of 50 nm/min with the bandwidth and time–response parameters set to 1 nm and 2 s, respectively.

### 3.9. Atomic Force Microscopy (AFM)

AFM measurements were performed in tapping mode by using a Multimode Nanoscope V system (Digital Instrument, Veeco, Plainview, New York, USA) using single-beam silicon cantilever probes (Bruker RFESP-75 0.01–0.025 Ohm-cm Antimony (n) doped Si, cantilever f0, resonance frequency 75 kHz, and constant force 3 N m^−1^). Peptides were dissolved in distilled water (GIBCO^®^) a day prior to imaging. AFM images were taken by depositing a 5 μL drop of peptide solution (final concentration of 0.01% *w*/*v*) onto freshly cleaved mica. Then, the EDC/sulfo–NHS solution was pipetted onto the top surface of the peptide solution. The sample was allowed to dry under ambient conditions for 5 min. Subsequently, samples were rinsed with distilled water to remove loosely bound peptides and unreacted EDC/sulfo–NHS, and then they were dried under ambient conditions for 30 min. The images were analyzed and visualized using the Nanoscope Software as previously described [19]. The morphological parameter analysis of the AFM data was performed using the Matlab-based open-source software FiberApp [61].

## 4. Conclusions

Here, we reported the design of cross-linked SAPs via a one-pot and in situ gelation system based on EDC/sulfo–NHS coupling, yielding self-healing and functionalized hydrogels. EDC/sulfo–NHS cross-linked SAP also exhibited an unusual two-step shear strain–stress behavior strictly correlated to the EDC/sulfo–NHS coupling.

Furthermore, we extended the scope of the EDC/sulfo–NHS coupling for the post-assembly functionalization of SAP nanofibers. Bioactive peptides thoroughly decorated the hydrogels without compromising their self-assembly propensity, molecular packing, and biomechanics. Our results point out that EDC/sulfo–NHS post-assembly cross-linking could be a useful tool to tailor the bioactivity and biomechanics of supramolecular nanostructures.

Overall, our approach may offer new additional tools to optimize the design and biomimetic properties of peptide biomaterials for biomedical applications independently from other key variables of supramolecular peptide hydrogels such as nanotopography and porosity.

## Supplementary Materials

Supplementary Materials can be found at https://www.mdpi.com/1422-0067/21/12/4261/s1.

## Abbreviations

SAPs	Self-assembling peptides
ECM	Extracellular matrix
EDC	1-ethyl-3-[3-dimethylaminopropyl]carbodiimide
Sulfo–NHS	N-hydroxysulfosuccinimide
TBI	Traumatic brain injury
SCI	Spinal cord injury
LVR	Linear viscoelastic regime
DMF	Dimethylformamide
HBTU	*N,N,N′,N′*-Tetramethyl-O-(1H-benzotriazol-1-yl)uronium hexafluorophosphate
DIEA	*N,N*-Diisopropylethylamine
NMP	*N*-Methyl-2-pyrrolidone
TFA	Trifluoroacetic acid
TIS	Triisopropylsilane
ThT	Thioflavin-T
FT-IR	Fourier transform infrared spectroscopy
CD	Circular dichroism
AFM	Atomic force microscopy
DPBS	Dulbecco’s phosphate-buffered saline
FITC	Fluorescein Isothiocyanate
DMSO	Dimethyl sulfoxide
