# Supplementary material for: Cross-Linked Self-Assembling Peptides and Their Post-Assembly Functionalization via One-Pot and In Situ Gelation System
Source: Int J Mol Sci. 2020 Jun 15;21(12):4261. doi: 10.3390/ijms21124261 (PMC7353005; doi:10.3390/ijms21124261)
Supplement: Supplementary file 1 [file ijms-21-04261-s001.pdf]

Table of Contents

Supplementary Figures **2**

Supplementary Figure S1: EDC/sulfo-NHS cross-linked LDLK12 peptide 2

Supplementary Figure S2: Shear stress (σ) measurements 2

Supplementary Figure S3: CD spectrum of EDC/sulfo-NHS 3

Supplementary Figure S4: 2D heights interpolation map 3

Supplementary Figure S5: Nematic order parameter 4

Supplementary Figure S6: CD spectra of KLPGWSG and LDLK12_EDC/NHS_KLPGWSG 5

Supplementary Figure S7: FITC-KLPGWSG fluorescence intensity 5

Supplementary Figure S8: HPLC and LC-MS analyses of LDLK12, KLPGWSG and FITC-KLPGWSG 8

Supplementary Figure S9: HPLC and LC-MS analyses of cross-linked LDLK12 9

Supplementary Figure S10: HPLC and LC-MS analyses of LDLK12 post-assembly functionalization with KLPGWSG 10

References **10**

International Journal of
Molecular Sciences

Supplementary Figures

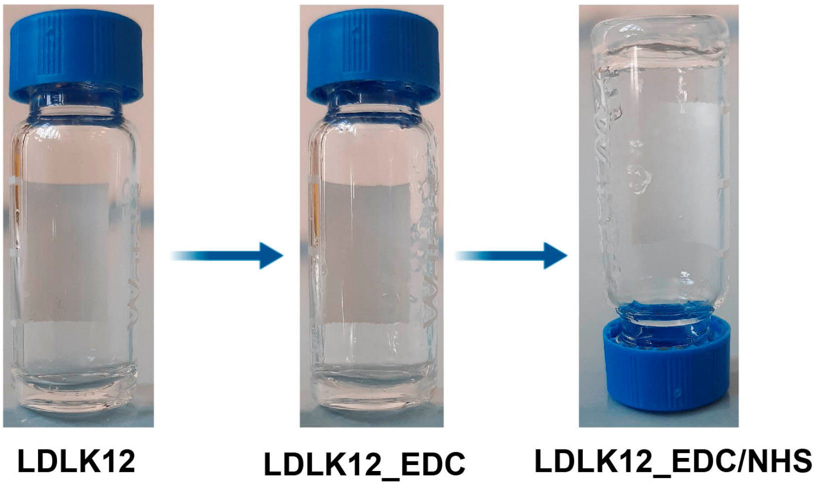

Supplementary Figure S1: EDC/sulfo-NHS cross-linked LDLK12 peptide. One-pot *in situ* LDLK12 (1% *w*/*v*) cross-linking via EDC/sulfo-NHS coupling: after cross-linking the self-supporting SAP scaffold can be appreciated.

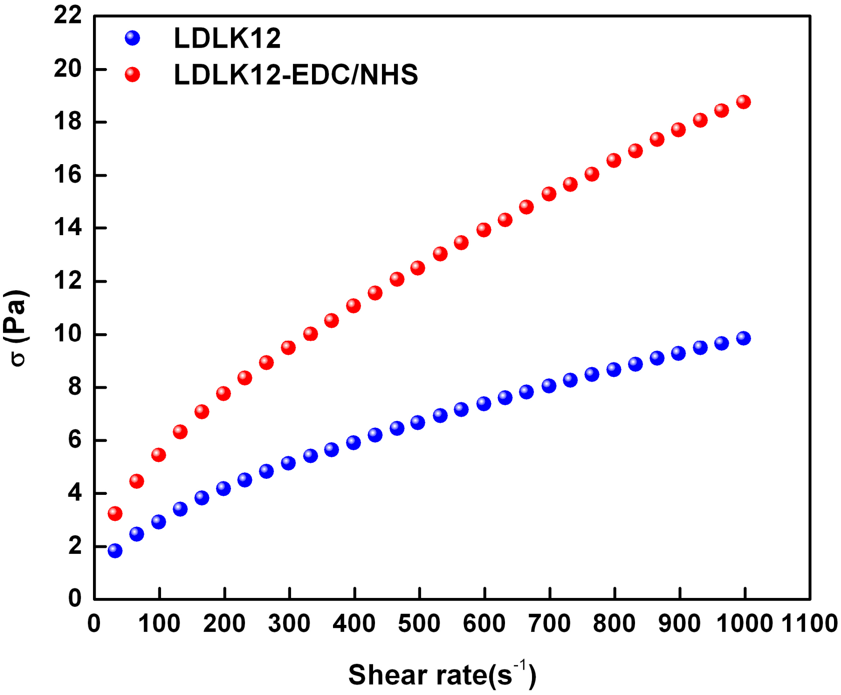

Supplementary Figure S2: Shear stress (σ) measurements. Shear stress (σ) measurements at increasing shear rate of the wildtype and cross-linked LDLK12 peptides. Both SAPs exhibited non-Newtonian shear-thinning behavior.

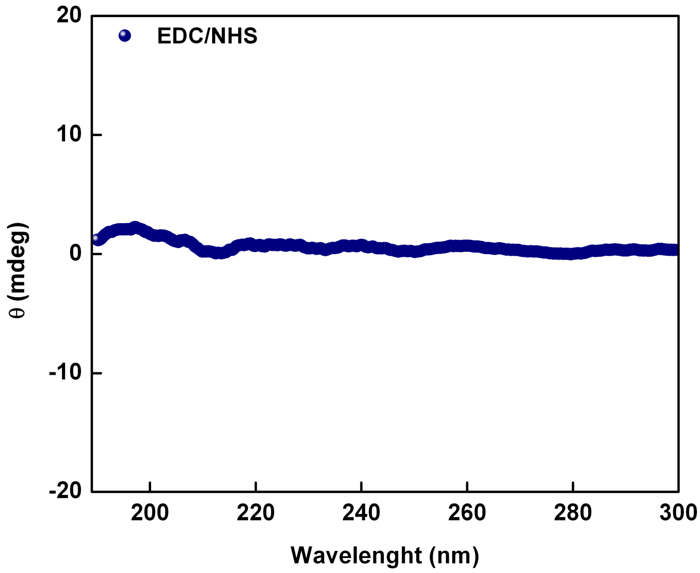

Supplementary Figure S3: CD spectrum of EDC/sulfo-NHS. No CD signal was observed in EDC/sulfo-NHS alone, suggesting no assembly propensity for the cross-linker alone.

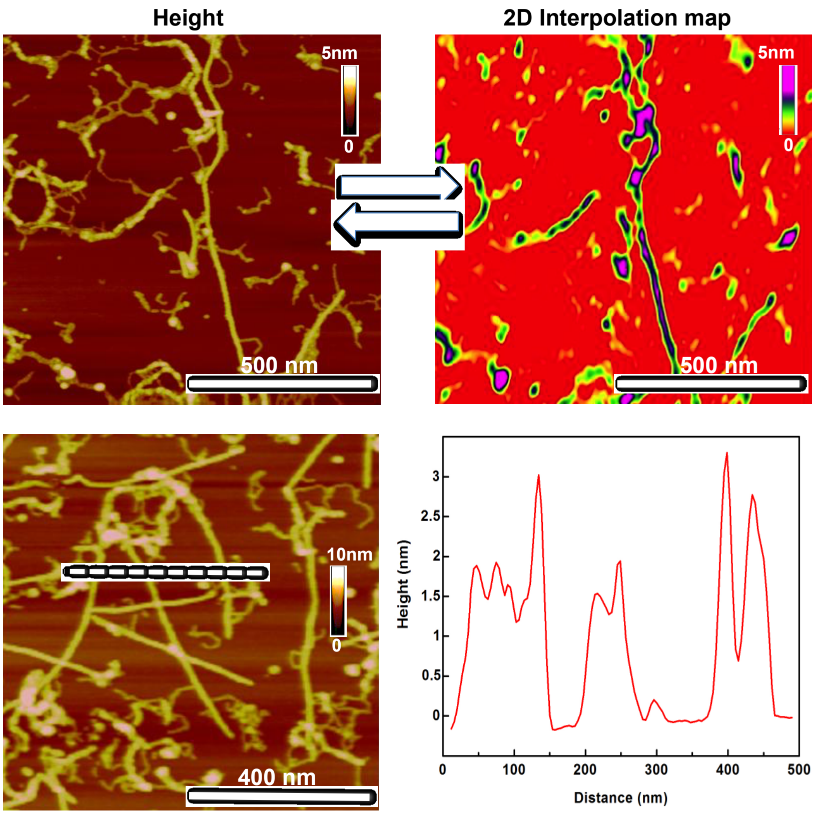

Supplementary Figure S4: 2D heights interpolation map. 2D heights interpolation map and height measurements of EDC/sulfo-NHS cross-linked LDLK12 peptide.

International Journal of
Molecular Sciences

MDPI

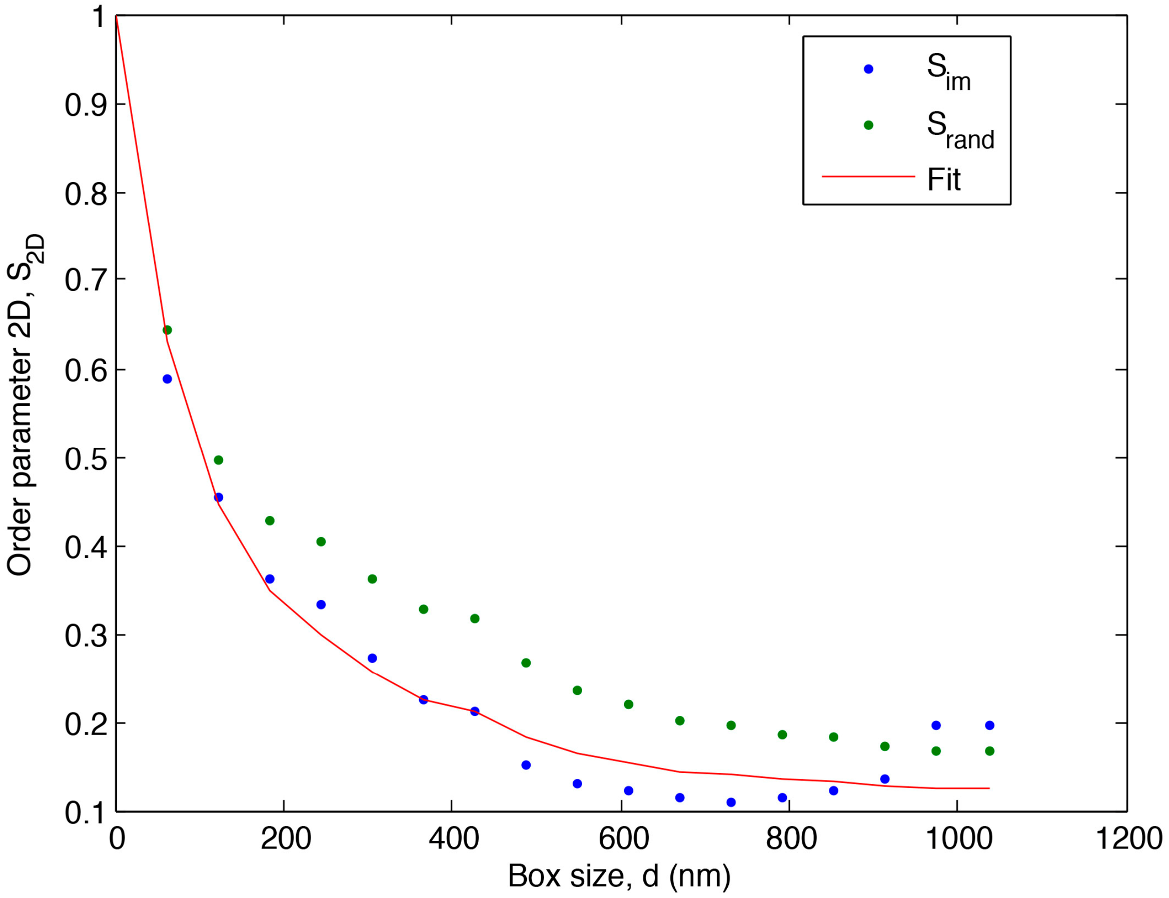

Supplementary Figure S5: Nematic order parameter. Nematic order parameter of cross-linked LDLK12 nanostructured scaffold, obtained by FiberApp software [60,61]. To calculate the 2D order parameter (i.e. S_{2D}) we divided the whole AFM image into square blocks of a certain size (*d*). Calculating and averaging S_{2D} values for all blocks results in one mean number, which is parametric with (*d*), yielding to the length scale-dependent $S_{2D}(d)$[62]. $S_{2D}(d)$ function is further expressed as sum of the weighted components S_{2D}^{align} and S_{2D}^{rand}, corresponding to the alignment of the nematic and isotropic components, respectively: $S_{2D}(d)= aS_{2D}^{align}(d) + (1 – a) S_{2D}^{rand}(d)$; where *a* is the relative surface fraction of the aligned (nematic) domains. By applying this analysis to the tracked nanofibrils, it is possible to quantify isotropic–nematic transitions rigorously.[61, 62].

International Journal of
Molecular Sciences

MDPI

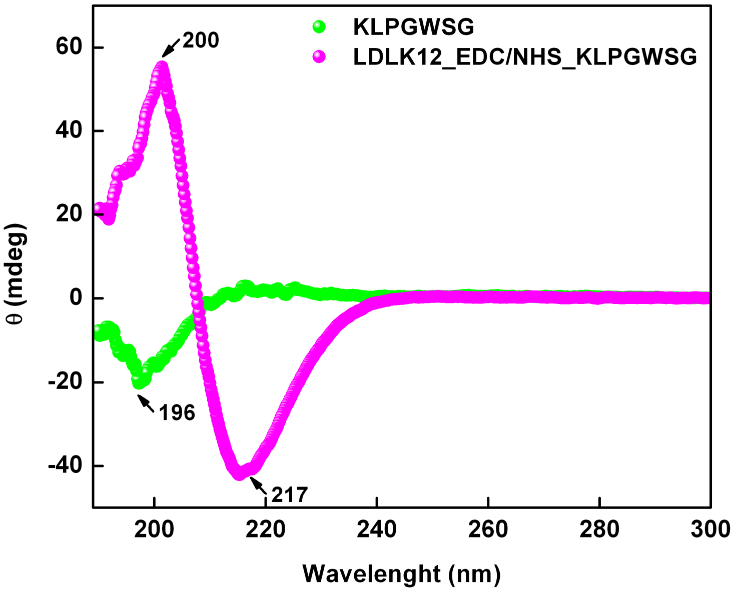

Supplementary Figure S6: CD spectra of KLPGWSG and LDLK12_EDC/NHS_KLPGWSG. LDLK12 peptide after the EDC/sulfo-NHS reaction with KLPGWSG showed the presence of β-sheet secondary structures, while the KLPGWSG alone showed an unstructured conformation.

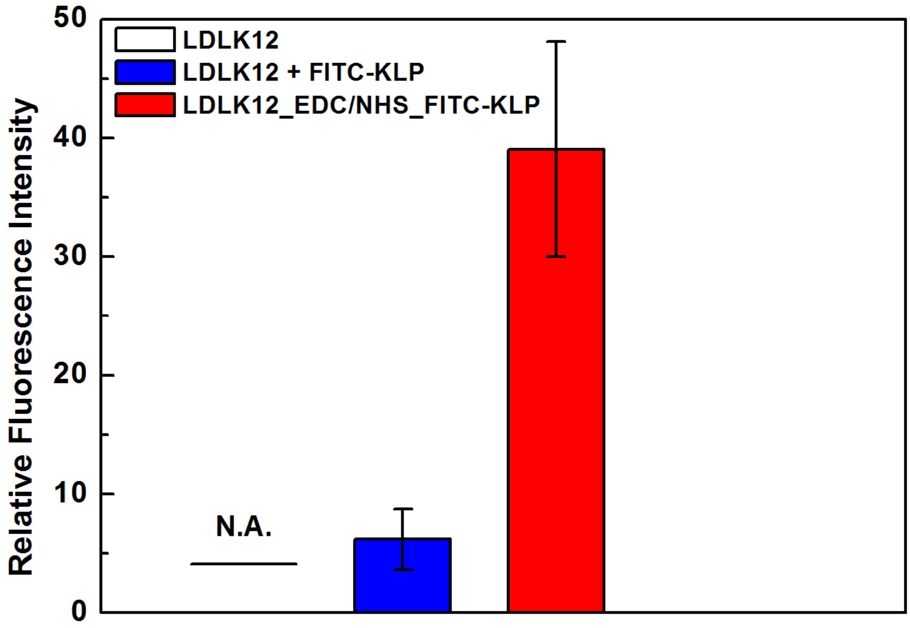

Supplementary Figure S7: FITC-KLPGWSG fluorescence intensity. Quantification of FITC-KLPGWSG fluorescence intensity on LDLK12 peptide nanostructures. EDC/sulfo-NHS-mediated conjugation of FITC-KLPGWSG peptide to LDLK12 nanofibers showed higher fluorescence intensity compared to the non-specific adsorption of FITC-KLPGWSG to nanofibers of standard LDLK12. As expected, LDLK12 alone did not show any detectable signal.

International Journal of Molecular Sciences

MDPI

Ac-LDLKLDLKLDLK-CONH$_2$

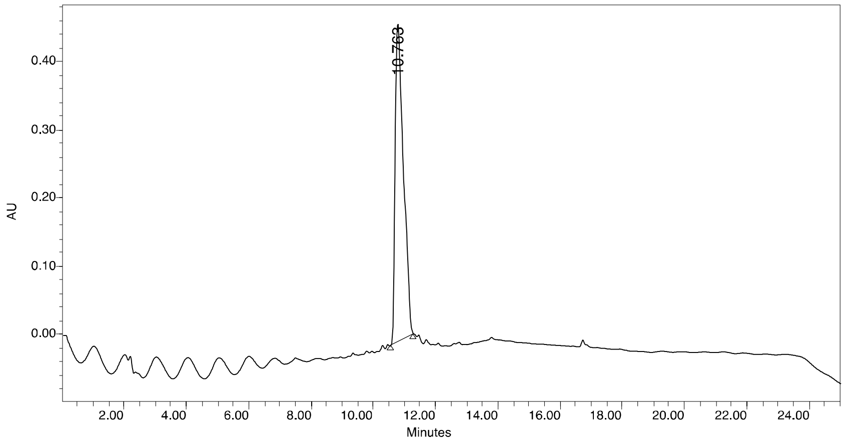

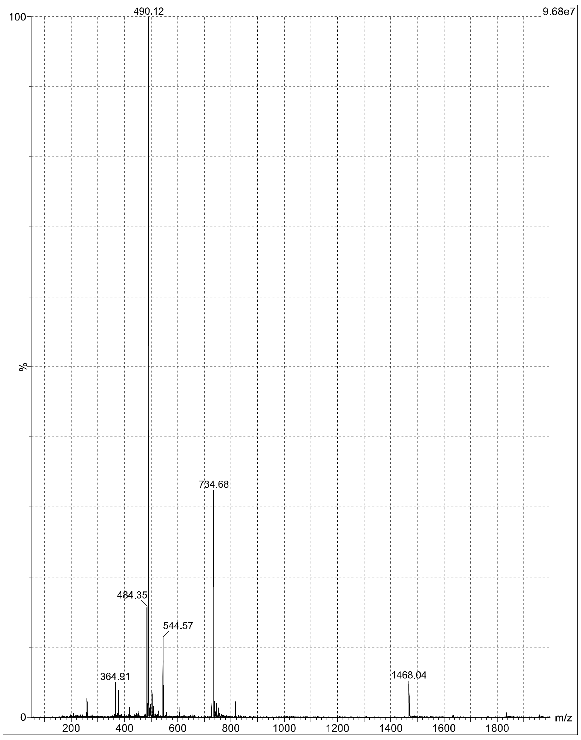

International Journal of
Molecular Sciences

H_2N-KLPGWSG-$CONH_2$

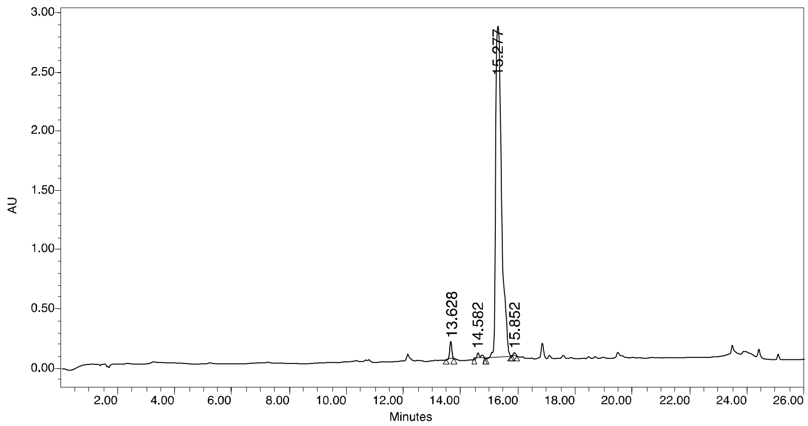

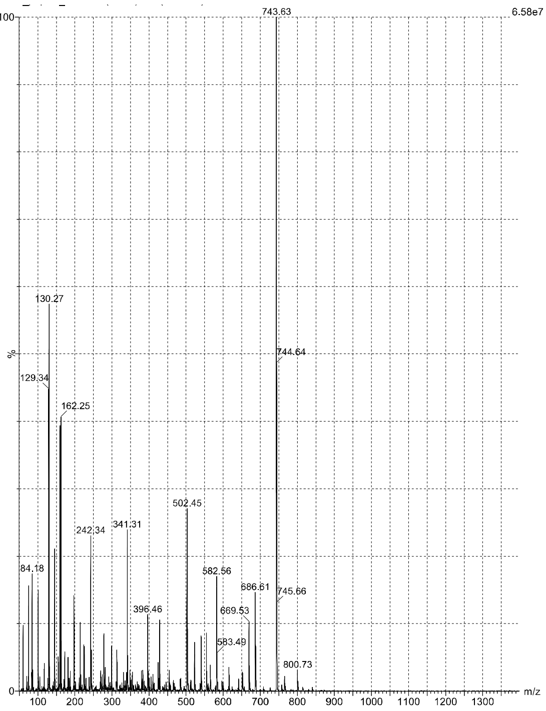

FITC-KLPGWSG-CONH$_2$

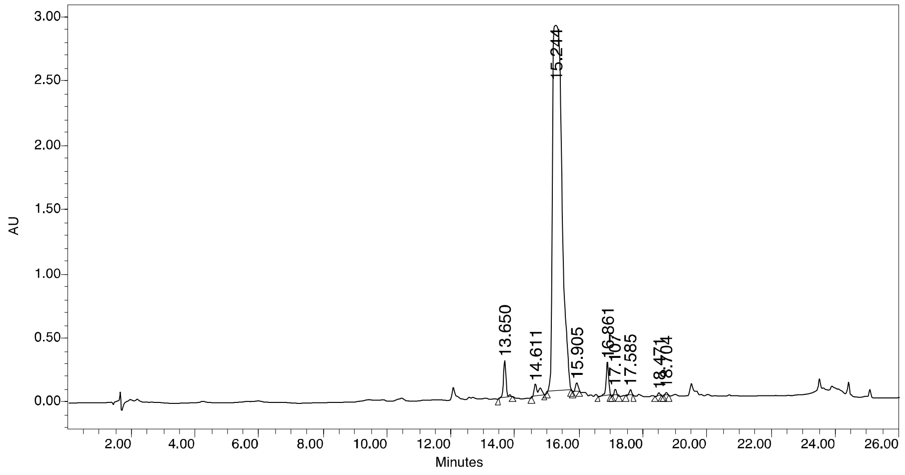

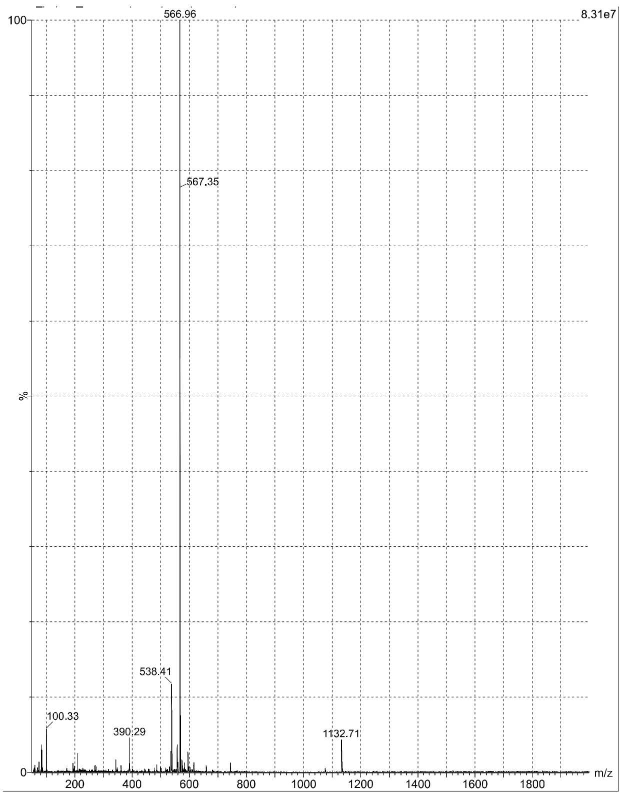

Supplementary Figure S8: HPLC and LC-MS analyses of LDLK12, KLPGWSG and FITC-KLPGWSG. LDLK12: LC-MS calc. = 1468.89 g/mol, obs. = 1468.04 g/mol. KLPGWSG: LC-MS calc. = 744.4 g/mol, obs. = 743.63 g/mol. FITC- KLPGWSG: LC-MS calc. = 1133.32 g/mol, obs. = 1132.71 g/mol.

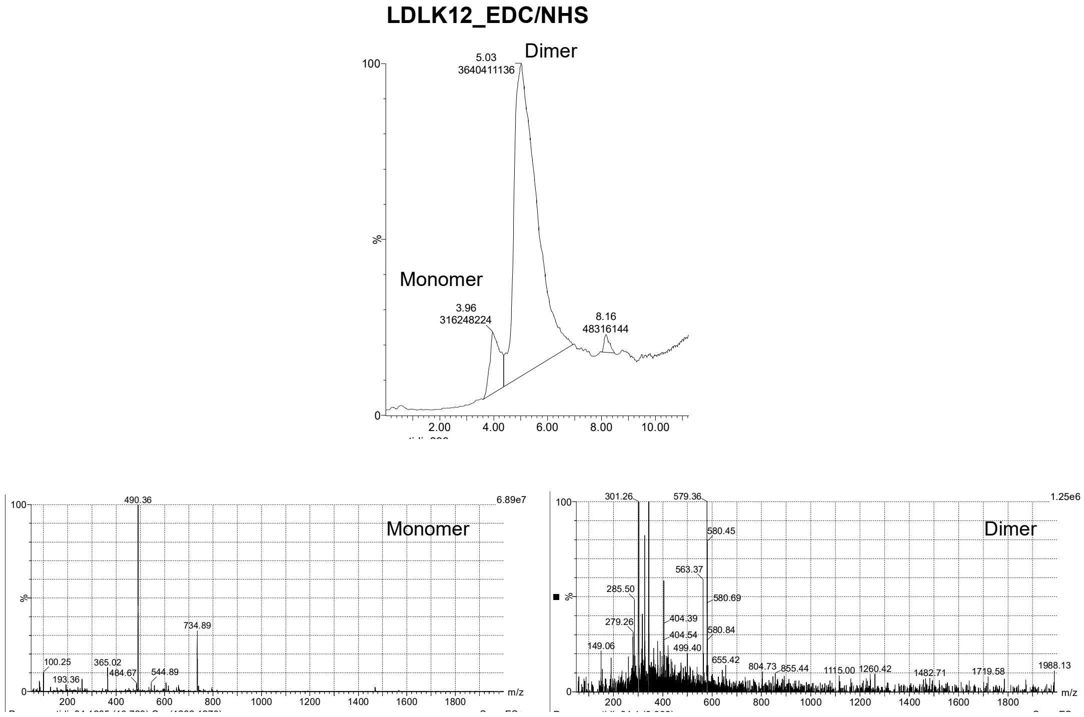

Supplementary Figure S9: HPLC and LC-MS analyses of cross-linked LDLK12. Monomer: LC-MS calc. = 734.95 g/mol, obs. = 734.89 g/mol. Dimer: LC-MS calc. = 2076 g/mol [1M+1H]+1, 1438 g/mol [1M+2H]+2 obs. = 1988.13 g/mol, 1482 g/mol. .

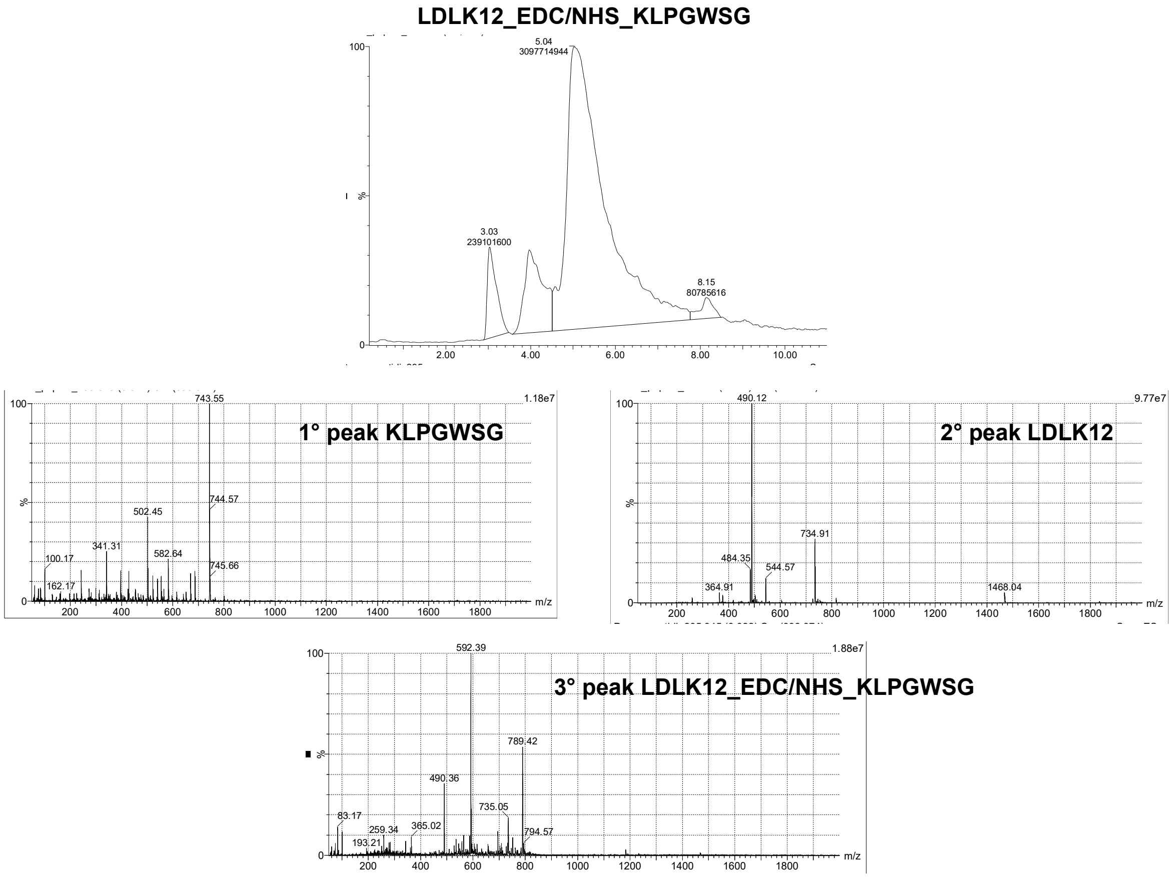

Supplementary Figure S10: HPLC and LC-MS analyses of LDLK12 post-assembly functionalization with KLPGWSG. 1°peak (KLPGWSG) LC-MS calc. = 744.4 g/mol, obs. = 743.55

g/mol; 2° peak (LDLK12) LC-MS calc. = 1468.89 g/mol, obs. = 1468.04 g/mol.; 3° peak (LDLK12_EDC/sulfoNHS_KLPGWSG) LC-MS calc. = 789.77 [1M + 4H]+ 4 g/mol, obs. = 798.42 g/mol.

Reference

60. Jordens, S.; Schwenke, K.; Usov, I.; Del Gado, E.; Mezzenga, R. Nematic field transfer in a two-dimensional protein fibril assembly. Soft Matter 2016, 12, 1830–1835.
61. Usov, I.; Mezzenga, R. FiberApp: An Open-Source Software for Tracking and Analyzing Polymers, Filaments, Biomacromolecules, and Fibrous Objects. Macromolecules 2015, 48, 1269–1280.
62. Jordens, S.; Isa, L.; Usov, I.; Mezzenga, R., Non-equilibrium nature of two-dimensional isotropic and nematic coexistence in amyloid fibrils at liquid interfaces. *Nature Commun.* **2013,** *4*, 1917.
